# Editorial for the Special Issue “Vasorum Lymphaticorum: From the Discovery of the Lymphatic System to the New Perspectives in Microsurgical Reconstruction and Patient Rehabilitation”

**DOI:** 10.3390/medicina60020307

**Published:** 2024-02-10

**Authors:** Alberto Bolletta, Luigi Losco, Emanuele Cigna

**Affiliations:** 1 Plastic Surgery and Microsurgery Unit, Department of Translational Research and New Technologies in Medicine and Surgery, University of Pisa, 56126 Pisa, Italy; 2 Plastic Surgery Unit, Department of Medicine, Surgery and Dentistry, University of Salerno, 84081 Salerno, Italy

Lymphedema is a complex clinical condition that appears as a result of the failure of the lymphatic system function, and it is characterized by edema, fibrosis, and adipose deposition. It is estimated that lymphedema affects up to 250 million people worldwide [1,2].

Over the past decade, a plethora of studies on lymphedema biology and surgical and conservative techniques have materialized as a testament to intense research but also as evidence of a few overall certainties [3,4,5].

In the early stages of the pathological process, conservative and nonsurgical treatment can be advised, but in more advanced stages, surgical treatment becomes necessary [6,7,8]. Early proposed surgical procedures were often invasive and disfiguring. In contrast, recently described surgical techniques have proved to be less invasive and yet still effective in reducing limb volume together with the risk of complications, like cellulitis, or the need for successive treatment; moreover, the recent trend of combining physiological and excisional procedures is definitely taking the stage [9,10,11].

This Special Issue aims to discuss the current trends in the management of lymphedema both in terms of conservative and surgical treatment.

We also showcase several studies focusing on the anatomy of the lymphatic network, as well as the molecular and physiological control of lymphatic vessel function and prophylactic approaches to the prevention of lymphedema [12].

In lymphedema patients, the goal of conservative treatment is controlling the swelling to restore the functionality of the affected limb and prevent potential complications. External compression is the mainstay of conservative treatment since compression therapy alone can achieve a limb volume reduction of as much as 60% [13,14]. Surgical intervention may be needed when conservative treatment does not lead to satisfying results. The comprehensive review performed by de Sire et al. [15] shows that several limitations affect the evidence supporting a specific surgical and rehabilitation treatment and how rehabilitation should be considered the cornerstone of the lymphedema treatment, not only for patients not suitable for surgery but also before and after surgical procedures. Moreover, a study by the same authors describes how multiple treatments such as manual lymphatic drainage (MLD) and intermittent pneumatic compression (IPC) may be used in a synergistic treatment and play a role in reducing lower limb lymphedema [16].

In lymphedema treatment, the correct timing for surgery is crucial, and super-microsurgical lymphaticovenular anastomosis (LVA) is a safe and effective procedure, which is particularly performed in early-stage cases when conservative therapies are insufficient to control the swelling [17,18]. The study performed by Caretto et al. [19] on planning LVA in three areas of the lower limb in early-stage gynecological cancer-related lymphedema (GCRL) secondary to pelvic lymphadenectomy reveals interesting results. The authors conclude that the placement of incision sites in all the anatomical subunits of the lower limb is one of the key factors in achieving good results in lower extremity lymphedema patients.

Lymphedema management becomes even more difficult and obscure in cases of unusual presentations, such as genital lymphedema (GL) [20]. Kaciulyte et al. [21] present the results of a treatment algorithm after over 38 years of experience, combining complete decongestive therapy (CDT), LVA, and excisional procedures.

The concept of a preventive approach to lymphedema was suggested by Boccardo et al. in 2009 [22]. In this study, the authors performed LVA at the time of axillary dissection, and lymphedema was prevented in all treated cases; several other preventive approaches to lymphedema have been described since.

Nacchiero et al. [23] elaborate on their experience in the prevention of secondary lymphedema after complete lymph node dissection (CLND) in melanoma patients by performing preventive lymphaticovenous anastomoses. The results of their study show how LVA significantly reduces the frequency of secondary lymphedema both in immediate and delayed CLND. Another approach is presented by Pierazzi et al., which involves the use of distally prophylactic lymphaticovenular anastomoses after axillary or inguinal complete lymph node dissection followed by radiotherapy [24]. In this case series, the authors show how performing LVA distally to the irradiated area after axillary or groin lymphadenectomy, as well as after adjuvant radiotherapy, ensures the long-term patency of anastomoses to reduce the risk of iatrogenic lymphedema.

Prophylactic treatment for lymphedema prevention has been also suggested in sarcoma resections. Wan et al. [25] suggest performing LVA procedures after resections of sarcomas of the lower limb at the level of the medial thigh and medial and lateral calf.

On the other hand, Scaglioni et al. [26] describe a different approach as they resorted, in all cases, to the superficial circumflex iliac artery perforator (SCIP) flap, in either its pedicled or free form, and to its lymphatic network, to perform lymphatic tissue transfer. In their study, they conclude that a reconstructive procedure that aims not only to restore the missing volume but also the lymphatic drainage might successfully reduce the rate of postoperative complications.

In summary, this Special Issue presents the latest evidence concerning the prevention, conservative, and surgical treatment of lymphedema. An increasing number of studies on the topic show how the treatment of this condition requires a multidisciplinary and tailored approach [6,27,28].
